# Extracts from *Cordyceps cicadae* and *Hericium erinaceus* promote the neurite outgrowth of retinal ganglion cells

**DOI:** 10.1371/journal.pone.0342244

**Published:** 2026-02-06

**Authors:** Fang-Yi Chen, Chin-Chu Chen, Chuan-Chin Chiao

**Affiliations:** 1 Institute of Molecular Medicine, National Tsing Hua University, Hsinchu, Taiwan; 2 Biotech Research Institute, Grape King Bio, Pingzhen, Taiwan; 3 Institute of Food Science and Technology, National Taiwan University, Taipei, Taiwan; 4 Department of Food Science, Nutrition, and Nutraceutical Biotechnology, Shih Chien University, Taipei, Taiwan; 5 Institute of Systems Neuroscience, National Tsing Hua University, Hsinchu, Taiwan; 6 Department of Life Science, National Tsing Hua University, Hsinchu, Taiwan; Advanced Materials Technology Research Institute, National Research Centre, EGYPT

## Abstract

The regenerative capacity of mammalian RGC neurites after damage, such as glaucoma, is limited. Numerous studies have utilized herbal extracts to promote neural regeneration and exert neuroprotective effects in the hope of mitigating glaucoma. In the present study, we investigated the effect of extracts from the cicada fungus (*Cordyceps cicadae*) and the Lion’s mane mushroom (*Hericium erinaceus*) on neurite outgrowth of retinal explants and isolated RGCs. We also examined whether these extracts affect the number of apoptotic cells and neurite outgrowth activity of RGCs. The results showed that an aqueous extract of *Cordyceps cicadae*, an ethanol extract of *Hericium erinaceus*, and the purified compound Erinacine Sare able to promote neurite outgrowth in retinal explants. Given its role as a key bioactive compound, Erinacine S was further investigated on isolated RGCs, where it also significantly enhanced neurite outgrowth, demonstrating a direct effect on RGC regeneration. In addition, these extracts have no significant drawbacks in terms of cell apoptosis and RGC neurite outgrowth activity at specific concentrations. The present study thus demonstrates that while excessively high concentrations of these extracts may inhibit neurite growth, at moderate concentrations some extracts from *Cordyceps cicadae* and *Hericium erinaceus* have the potential of promoting neurite regeneration in the mammalian retina. Further research targeting the molecular mechanisms behind these effects may shed light on their potential application as a medicine or nutraceutical for facilitating neural regeneration.

## Introduction

The regenerative capacity of the central nervous system (CNS) in mammals gradually declines after the organism reaches its maturity [1]. Both intrinsic and extrinsic factors are known to influence the limited regenerative capacity of CNS neurons. Extrinsically, it is primarily attributed to the formation of regenerative barriers by specific types of cells. For instance, glial cells create physical barriers that prevent the penetration of axons at the site of injury, and can also release inhibitory molecules that restrict synaptic growth; intrinsic changes during early development have been shown to reduce axon regeneration potential [2, 3]. The mammalian retina, which is part of the CNS, contains photoreceptors responsible for receiving light signals and transmitting these to the downstream retinal ganglion cells (RGCs). The RGCs then transmit visual information, mainly through the lateral geniculate nucleus (LGN), to the visual cortex of the brain [4]. Therefore, when RGCs are damaged, axon regeneration becomes difficult, which consequently disrupts the transmission of visual information to the brain. Among eye diseases, glaucoma is the most common neurodegenerative disease worldwide [5]. Glaucoma is characterized by abnormal secretion of aqueous humor, which is produced by the ciliary body, and this results in an elevated intraocular pressure [6]. This elevated pressure compresses the retinal cells, which leads to damage and degeneration of the RGCs and the optic nerve. As a result, patients’ vision is affected and the patient can become blind permanently. Therefore, promoting neurite outgrowth in in vitro RGC models is considered a critical initial step for assessing neurite regeneration in vivo and the potential for optic nerve repair following glaucoma-induced injury.

There are numerous studies investigating the neuroprotective and neural regenerative effects of herbal extracts [7, 8]. For example, Ginkgo (*Ginkgo biloba*) extract, known for its antioxidant properties, has been shown to reduce cellular oxidative stress, and has therapeutic potentials for the treatment of Parkinson’s disease and Alzheimer’s disease [9]. Extracts from the petals of the Peony (*Paeonia lactiflora*) have been shown to exhibit both antioxidant activity and neurite outgrowth activity, indicating that they have both neuroprotective and regenerative effects [10]. In addition, there are some studies that support the effects of herbal extracts on enhancing the neural regeneration of retinal cells. For instance, a mixture of American ginseng (*Panax quinquefolius L.*) extract, *Ginkgo biloba* extract, and St John’s wort (*Hypericum perforatum*) extract has been shown to increase the number of regenerated RGCs [11]. Tiger milk mushroom (*Lignosus rhinocerotis*) and Lion’s mane mushroom (*Hericium erinaceus*) aqueous extracts have also been shown to promote the neurite outgrowth of neurons in the brain, spinal cord, and retina [12]. *Hericium* family and *Hericium*-derived compounds have been widely investigated for their diverse biological and pharmacological properties, particularly their neuroprotective and neurotrophic effects, immunomodulatory activity, anti-cancer potential, antioxidant capacity, and antimicrobial actions [13–15]. In some studies, *Hericium enrinaceus* has shown anti-neuroinflammatory in cell culture [16–18].

Cicada fungus (*Cordyceps cicadae*), also known as “Da Chan Hua”, is a traditional Chinese medicinal herb and tonic food. It is a fungus that parasitizes the larvae or nymphs of *Cicada flammate*, absorbing nutrients from the host to form a biological complex of larvae and fungus [19]. Previous studies have shown that extracts from *C. cicadae* are able to enhance immune responses, possess anti-tumor properties, and exhibit antioxidant characteristics [20]. For instance, polysaccharides derived from *C. cicadae* have been found to reduce intracellular oxidative stress and increase cell viability [21]. A recent study has also revealed that *C. cicadae* mycelium extract is able to significantly reduce the elevated intraocular pressure caused by glaucoma [22]. Another extract, N6-(2-Hydroxyethyl) Adenosine (HEA), has shown effectiveness in various models of eye diseases. Previously published results show that HEA has the ability to decrease intraocular pressure, relieve dry eye symptoms, and reduce ultraviolet (UV)-induced cataract development [23–25]. However, there is limited research on the effects of *C. cicadae* mycelium extracts and HEA on neuronal regeneration. Their potential on neurite outgrowth and neuronal regeneration remains largely unexplored, providing a rationale for their inclusion in the present study.

Lion’s mane mushroom (*Hericium erinaceus*), commonly known as “Monkey Head Mushroom”, belongs to the *Hericium* genus in the *Hericiaceae* family of the *Basidiomycota* phylum. This edible mushroom contains bioactive compounds, including polysaccharides, hericenones, and erinacines. Among the above, erinacines consist of a series of compounds that are classified as A-S [26]. Previous studies have shown that ethanol extracts of *H. erinaceus* exhibit nerve growth factor (NGF)-inducing activity in 1321N1 human astrocytoma cells [27]. Erinacine A and Erinacine S have been found to promote the maturation of oligodendrocytes and seem to have potential for myelination in neurodevelopmental disorders [28]. Furthermore, it has been reported that Erinacine S is able to penetrate the blood-brain barrier in rats, suggesting that there is potential for *H. erinaceus* extracts to be used in the treatment of neurological diseases [29]. Taken together, these findings provide a biological rationale for investigating the effects of *H. erinaceus* extracts and erinacines on neurite outgrowth in retinal ganglion cells. In the present study, we examined the effects of *C. cicadae* ethanol extract (*Cordyceps cicadae*-EtOH), *C. cicadae* aqueous extract (*Cordyceps cicadae*-H_2_O), N6-(2-Hydroxyethyl) Adenosine (HEA) from *C. cicadae* mycelium, *H. erinaceus* ethanol extract (*Hericium erinaceus*-EtOH), Erinacine A from *H. erinaceus* and Erinacine S from *H. erinaceus* on the promotion of neurite outgrowth of retinal explants and isolated RGCs from postnatal mice. We applied these extracts using different concentrations to assess the effective range of enhanced neural regeneration. We also examined whether these extracts had any impact on cell apoptosis and the neurite outgrowth activity of RGCs.

## Materials and methods

### Preparation and culture of retinal explants

Retinas were isolated from C57BL/6 mice at postnatal day 8 (P8), and there were at least three batches of mice with different birth dates. All experimental procedures adhered to the guidelines stipulated by the Institutional Animals Care and Use Committee of the National Tsing Hua University (Protocol Number: #110014) and were consistent with the ARVO Statement for the Use of Animals in Ophthalmic and Vision Research.

Anesthesia was initiated by administering isoflurane through inhalation (NDC: 66794−013, Piramal Critical Care, Bethlehem), in combination with an intramuscular injection of an excessive dose of 10 mg/kg Ketamine (NDC: 08443, Balanzine, Taiwan) and 10 mg/kg Xylazine (NDC: 05417, IMALGENE1000, Taiwan) to ensure humane euthanasia. All procedures were performed to minimize pain and distress.

Utilizing dissecting scissors, the eyelid was incised, and the eyeballs were extracted using forceps before being immersed in oxygenated Ames’ medium (A1420; Sigma-Aldrich) supplemented with 23 mM NaHCO_3_. Subsequently, the lens was excised, and the retina delicately separated from the retinal pigment epithelium. Following meticulous removal of vitreous humor with Dumont forceps, each retina was individually isolated and then sectioned into four pieces. Each piece was treated with the corresponding concentration of the extract, which was considered as one sample. These peripheral retinal explant segments were then positioned on 18-mm coverslips (0111580; MARIENFELD) coated with Cell-Tak (354240; CORNING), with the ganglion cell side facing downwards, before proceeding with the culture process outlined below.

**Fig 1:**
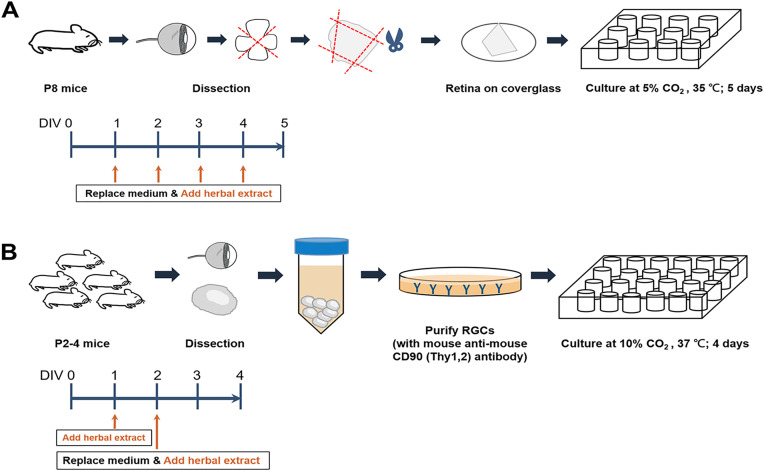
Flowcharts of retinal explant and retinal ganglion cell cultures. **(A)** Retinas were isolated and divided into four pieces. The retinal explants were then cultured in a 12-well plate with the medium being replaced daily and the herbal extract added each day. **(B)** Retinas were isolated and collected in a tube. RGCs were purified via immunopanning and subsequently cultured in a 24-well plate. The herbal extract was added at Day 1 (DIV 1) in vitro. At DIV 2, half of the fresh culture medium was replaced and the herbal extract was added.

The retinal explants, situated on coverslips, were subsequently transferred into a 12-well culture plate and maintained in a humidified incubator with 5% CO_2_ at 35 °C for a duration of 5 days (Fig 1A). Fresh culture medium was replenished daily into each well of the culture plate. The composition of the culture medium comprised Neurobasal-A (10888; GIBCO), 0.6% Glucose (G7528; SIGMA), 1X B-27 (17504−044; GIBCO), 1 mM Sodium Pyruvate (11360−070; GIBCO), 2 mM L-Glutamine (25030081; GIBCO), 10 mM HEPES (15630−080; GIBCO), 100 μg/mL Penicillin/Streptomycin (15140−122; GIBCO), 2.5 μg/mL Insulin (91077; SIGMA), 6 mM Forskolin (F6886; SIGMA), and 0.01 μg/mL IGF-1 (cyt-216; Prospec). The culture condition of 5% CO₂ for retinal explants was selected based on established protocols for maintaining tissue viability in organotypic retinal cultures [30]. The culture medium used is already optimized to support baseline neurite outgrowth in retinal explants, providing a reference level for assessing the effects of the tested extracts.

### Purification and culture of retinal ganglion cells

Two 150x10 mm petri dishes (351058; FALCON) coated with 60 μL Goat anti-rabbit IgG (111-005-003; Jackson) in 20 mL Tris-HCl (50 mM, pH 9.5; T-1503; SIGMA) and one 100x10 mm petri dish (351029; FALCON) coated with 30 μL Goat anti-mouse IgM (115-005-020; Jackson) in 12 mL Tris-HCl were prepared in advance. All of the coated petri dishes were incubated at 4 °C overnight. The 100x10 mm coated petri dish was washed with DPBS twice before sacrificing the mice. Finally, 70 μL Mouse anti-mouse Thy1 clone F7D5 (MCA02R; BIO-RAD) and 10 mL 1X DPBS (14287−072; GIBCO) was added into the dish, which was then incubated at 4 °C for at least two hours. In addition, 12 mm coverslips coated with Poly-D-Lysine (50 μg/mL; A38904-01; GIBCO) were prepared and placed in each of the 24 wells. The 24-well plates were then incubated at 37 °C overnight. Next day, the coated coverslips were washed with sterile ddH_2_O, and then coated with Laminin (10 μg/mL; 23017−015; Invitrogen) by incubating at 37 °C for at least eight hours.

A retina was dissected from postnatal days 2−4 (P2-4) C57BL/6 mice (Fig 1B). More than 14 mice were used in each experiment. Animals were anesthetized via inhalation of isoflurane and administered an intramuscular overdose of a 1:1 mixture of Ketamine and Xylazine to ensure humane euthanasia. All procedures were performed to minimize pain and distress. The eyeballs were then extracted using forceps and transferred to oxygenated (95% O2 and 5% CO2) 1X DPBS. Next 40−45 μL of papain (9001734; SIGMA) and 5 μL of 1 M NaOH (3722−19; J.T. Baker) were added to 10 mL of 1X DPBS. The detached retinas were added into this solution. To dissociate the retinal tissues, the solution was subjected to incubation in a 37 °C water bath for 30 minutes with shaking every 15 minutes. The liquid was removed and the remaining tissue was then transferred to a buffer medium containing Rabbit anti-mouse macrophage (AIAD31240; Accurate Chemical). This was poured into a 150x10 mm petri dish coated with Goat anti-rabbit IgG and incubated for 20 minutes with shaking every 10 minutes. Then, the solution was poured from the first 150x10 mm petri dish into the second 150x10 mm petri dish containing Goat anti-rabbit IgG and incubated for 45 minutes with shaking every 15 minutes. Finally, the solution was poured from the second petri 150x10 mm dish into the third 100x10 mm petri dish coated with Goat anti-mouse IgM and Mouse anti-mouse Thy1 clone F7D5 and incubated for 50 minutes with shaking every 10 minutes.

Retinal ganglion cells bound to the Thy1 coating were released by treatment with 2.5% trypsin (T4665; SIGMA) and 30% FBS (A3160501; GIBCO) and collected from the bottom of the dish. The collected cells were then centrifuged at 80 g for 15 minutes. After removing the supernatant, the cells were resuspended in culture medium and placed in a 24-well culture plate at a concentration of 5000 cells per well, and then cultured in 10% CO_2_ in a humidified incubator at 37 °C for 4 days. Each well of the culture plate had half of its volume replaced with fresh culture medium every two days. The 30 mL of culture medium contained 27.510 mL Neurobasal-A (10888; GIBCO), 300 μL 100X Pen/Strep, (15140122; GIBCO), 300 μL 100X Insulin (5ug/mL stock; 91077; SIGMA), 300 μL Sodium pyruvate (100 mM; 11360−070; GIBCO), 300 μL 100X Sato stock, 300 μL 100X Triiodo-thyronine (T6397; Sigma), 300 μL 100X L-Glutamine (200 mM; G8540; SIGMA), 30 μL 1000X N-acetyl L-cystein (A7250; SIGMA), 600 μL 50X B-27 (17504−044; GIBCO), 30 μL BDNF (50 μg/mL; 450−02; PEPROTECH), 30 μL CNTF (10 μg/mL; 450−13; PEPROTECH), 30 μL Forskolin (5 mM; F3917; SIGMA). Purified RGCs were cultured under 10% CO₂, which has been optimized in our laboratory and is consistent with previous studies reporting improved neuronal survival and neurite outgrowth under this condition [31]. The culture medium used is already optimized to support baseline neurite outgrowth in retinal ganglion cells, providing a reference level for assessing the effects of the tested extracts.

### Preparation of herbal extract

There were six stocks of herbal extracts and these were provided by the Grape King Bio Ltd. These six samples were all in powder form when received. The preparation protocols for these extracts are outlined below:

1. The ethanol extract of *Cordyceps cicadae* (Cc-EtOH). The fermented mycelium powder of *C. cicadae* was blended with 95% ethanol at a ratio of ten times the volume with ultrasonic for 60 minutes. After centrifugation, the supernatant was collected and concentrated under reduced pressure to obtain an ethanol extract. The extract powder was dissolved in sterile water containing 1% DMSO to prepare the stock solution, which was further diluted in the culture medium to achieve the desired final concentration of the extract, with the final DMSO concentration not exceeding 0.1%.
2. The aqueous extract of *Cordyceps cicadae* (Cc-H_2_O). The fermented mycelium powder of *C. cicadae* was mixed with RO water, also at a ratio of ten times the volume heating at 100°C for 30 minutes. After centrifugation, took the supernatant and concentrated under freeze-dried to obtain an aqueous extract. The extract powder was dissolved in sterile water as a stock solution, then added to the culture medium for the final treatment.
3. N6-(2-Hydroxyethyl) Adenosine (HEA) served as the standard (Sigma-Aldrich, St. Louis, MO, USA) for comparison with the *C. cicadae* extract using HPLC analysis conducted with an ultraviolet detector on the L-5000 Series system by Hitachi, Japan. The extract powder was dissolved in sterile water to prepare the stock solution, then added to the culture medium for the final concentration.
4. The ethanol extract of *Hericium erinaceus* (He-EtOH). The mycelium powder of *H. erinaceus* was mixed with 95% ethanol at a ratio of ten times the powder volume with ultrasonic for 60 minutes. After centrifugation, took the supernatant and concentrated under reduced pressure to obtain an ethanol extract. The extract powder was dissolved in sterile water with 0.8% DMSO to prepare the stock solution, which was then diluted into the culture medium to achieve the desired final concentrations of the extract, with the final DMSO concentration not exceeding 0.1%.
5. Extraction and purification of Erinacine A and Erinacine S from *H. erinaceus* were conducted in accordance with established methodologies from prior studies [32, 33]. The extract powders were dissolved in DMSO to prepare their respective stock solutions, which were then then diluted into the culture medium to achieve the desired final concentrations of the compounds, with the final DMSO concentration not exceeding 0.1%. Concentration selection was based on information provided by Grape King Bio Ltd. and exploratory testing with 10-fold dilutions.

### HPLC analysis of herbal extract

The herbal extracts underwent analysis using High-Performance Liquid Chromatography (HPLC) on an L-5000 Series system by Hitachi, Japan, equipped with an ultraviolet (UV) detector set to a wavelength of 254 nm (S1 and S2 Figs). The separation was achieved on a reverse-phase column (Inertsil^®^, ODS-2, 250 × 4.6 mm, 5 μm; GL Science, Tokyo, Japan) maintained at a column oven temperature of 40 °C. The mobile phase comprised 10 mM KH_2_PO_4_ and acetonitrile, with a linear gradient commencing at 96% KH_2_PO_4_. An injection volume of 10 μL was employed, and the flow rate was consistently set at 1.0 mL/min throughout the chromatographic analysis.

### Herbal extract treatment

A double-blind test was employed in the present study, whereby the composition and names of these samples were kept undisclosed until the completion of the experiment, at which point the blinding was lifted. The treatment scheme is outlined in Fig 1. The various treatments were:

1. N6-(2-Hydroxyethyl) Adenosine (HEA) from *C. cicadae*. The experimental concentrations were 1, 10, and 100 μg/mL (equivalent to 3.21 μM, 32.12 μM and 321.24 μM) in both retinal explant and retinal ganglion cell cultures.
2. The ethanol extract of *C. cicadae* (Cc-EtOH) was prepared and diluted using DMSO. The experimental concentrations were 1, 10, and 100 μg/mL in both retinal explant and retinal ganglion cell cultures.
3. The aqueous extract of *C. cicadae* (Cc-H_2_O). The experimental concentrations were 1, 10, and 100 μg/mL in both retinal explant and retinal ganglion cell cultures.
4. Erinacine A from *H. erinaceus* (HeA) was prepared and diluted using DMSO. The experimental concentrations were 0.05, 0.5, 5, and 10 μg/mL (equivalent to 115.61 nM, 1.16 μM, 11.56 μM, and 23.12 μM) in the retinal explant culture, and 0.05, 0.5, and 5 μg/mL in the retinal ganglion cell culture.
5. Erinacine S from *H. erinaceus* (HeS) was prepared and diluted using DMSO. The experimental concentrations were 0.05, 0.5, 5, and 10 μg/mL (equivalent to 116.14 nM, 1.16 μM, 11.61 μM, and 23.23 μM) in the retinal explant culture, and 0.05, 0.5, and 5 μg/mL in the retinal ganglion cell culture.
6. The ethanol extract of *H. erinaceus* (He-EtOH) was prepared and diluted using DMSO. The experimental concentrations were 1, 10, and 100 μg/mL in both retinal explant and retinal ganglion cell cultures.

Since there was no significant difference among untreated, water-treated, and DMSO-treated groups, the untreated group was designated as the negative control for comparison with extract-treated groups. A brief comparison confirmed that neither DMSO nor water treatments caused significant changes in retinal explant or retinal ganglion cell outgrowth compared with the untreated group (S3-S5 Fig).

### Immunofluorescence

Retinal explants under culture conditions were subjected to fixation using a solution consisting of 4% paraformaldehyde and 0.1% glutaraldehyde in 1X phosphate-buffered saline (PBS; C7001; ACE Biolabs) for a duration of 1 hour at room temperature. Following fixation, the explants underwent a triple rinse with 1X PBS, each rinse lasting 10 minutes. Subsequently, the fixed retinal explants were subjected to blocking using a mixture of 4% normal donkey serum (017-000-121; Jackson), 1% Triton X-100 (9002-93-1; SIGMA), and 0.1% sodium azide (S2002; SIGMA) in PBS for a period of 1 hour. Primary antibodies targeting the axonal marker beta-III-tubulin (TUJ1; 1:500; GTX631836; GeneTex) and cleaved caspase-3 (Asp175; 1:400; 9661S; Cell Signaling) were then applied to the retinal explants and allowed to incubate overnight at 4 °C. Post-incubation, the samples underwent a series of triple rinses with 1X PBS, each rinse lasting 20 minutes. Following this, the explants were incubated with the respective secondary antibody (1:250; CFR488A; BIOTIUM or 1:250; 711-025-152; Jackson) overnight at 4 °C. Subsequently, the explants were mounted onto slides using a mounting medium containing DAPI (H-1200; VECTASHIELD).

Primary retinal ganglion cells in culture were fixed by exposure to a solution comprising 4% paraformaldehyde and 0.1% glutaraldehyde in 1X phosphate-buffered saline for 20 minutes at room temperature. Post-fixation, the cells were subjected to a triple rinse with 1X PBS, each rinse lasting 10 minutes. Subsequently, the fixed cells were blocked using a mixture of 4% normal donkey serum, 0.2% Triton X-100, and 0.04% sodium azide in PBS for a duration of 1 hour. A primary antibody against beta-III-tubulin (TUJ1; 1:800; GTX631836; GeneTex) was then applied to the retinal ganglion cells and allowed to incubate overnight at 4 °C. Following incubation, the samples underwent a series of six rinses with 1X PBS, each rinse lasting 5 minutes. Post-rinsing, the cells were exposed to the secondary antibody (1:600; CFR488A; BIOTIUM) for 1 hour at room temperature. Finally, the explants were mounted onto slides using a mounting medium containing DAPI (H-1200; VECTASHIELD).

### Quantification of neurite outgrowth

**Fig 2:**
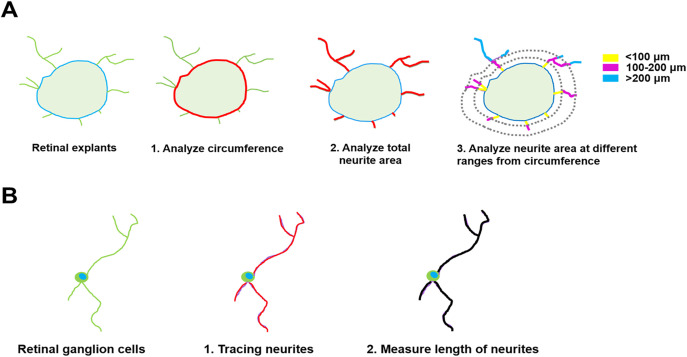
Schematic diagrams of the quantification of the neurite outgrowth of retinal explants and retinal ganglion cells. **(A)** The retinal explant images were split into two channels: blue (DAPI) and green (TUJ1). [1] The area of the retinal explant was defined using the blue channel, and the tissue perimeter was measured. [2] The total area of neurites expanded from the retinal explant was quantified. [3] The area outside the explant was divided into three regions: 100 μm (yellow), 100-200 μm (pink), > 200 μm (blue). Neurite areas grown out from the explants were measured in each of these regions. **(B)** The immunostained image of retinal ganglion cells consisted of blue (DAPI) and green (TUJ1) signals. [1] Neurite length was traced using Neuron **J.** [2] The length of grown neurite from the retinal ganglion cells was measured.

Neurite outgrowth from the retinal explants was captured utilizing a confocal microscope (LSM-800; Carl Zeiss) equipped with a 10X objective lens. To take complete images of the retinal explants, the z-stack and tiles functions of ZEN (blue edition) were used. The confocal images underwent segmentation into distinct color channels, where blue represented DAPI for the area of retinal explants, and green represented TUJ1 for the area of neurite growth. Quantification of neurites emerging from the explants was performed, defining the total neurite area using ImageJ (National Institute of Health, USA; Fig 2A). The evaluation of neurite outgrowth was determined by dividing the cumulative neurite area by the perimeter of the explants. Additionally, to distinguish neurites of different lengths originating from the explants, distinct assessments were performed for neurite areas categorized as “< 100 μm”, “100-200 μm”, and “> 200 μm” extending away from the explants’ boundary. This analytical methodology mirrors that employed by Gaublomme et al.[34] and Lee and Chiao [35].

Images of neurite outgrowth from the primary retinal ganglion cells were obtained utilizing an LSM-800 confocal microscope manufactured by Zeiss, equipped with a 10X objective lens. The acquired confocal images were segregated into distinct color channels: blue for DAPI, delineating the cellular soma, and green for TUJ1, depicting the region occupied by extended neurites. To calculate the average neurite lengths, ten cells were selected from a well randomly. Neurites grown out from the soma were quantified using NeuronJ (A plug-in program of ImageJ, NIH; Fig 2B).

### Quantification of neurite bearing cells

The center field was examined in each well of the incubated retinal ganglion cells using a LSM-800 confocal microscope (Zeiss). At least 30 cells were selected in the chosen field using ImageJ (NIH). The neurite-bearing cells, the neurite extension of which was double or more than the length of their soma diameter were counted. The proportion of cells possessing neurites was ascertained by dividing the tally of neurite-bearing cells by the overall cell count within the observed area.

### Cell apoptosis assay

The cultured retinal explants were imaged using an LSM-800 confocal microscope (Zeiss) equipped with a 10X objective lens, and stained with cleaved caspase-3. Following this, the confocal images were split into separate color channels: blue representing DAPI, marking the region of the retinal explants, and red indicating cleaved caspase-3, highlighting the area containing apoptotic cells. Quantification was performed solely on apoptotic cells exhibiting cleaved caspase-3 signal intensity surpassing a pre-established threshold. The ratio of apoptotic cells was calculated by dividing the area covered by apoptotic cells by the region stained with DAPI. All image processing procedures were carried out using ImageJ (NIH).

### Statistical analysis

The neurite outgrowth extents of the retinal explants and retinal ganglion cells under different herbal extract treatments were compared utilizing the non-parametric Kruskal-Wallis H test, followed by post hoc comparisons employing the Mann-Whitney U test with Bonferroni correction for multiple testing. All experimental data were expressed in mean ± SEM. Statistical analyses were performed using Excel (Microsoft; RRID: SCR_016137) and SPSS Statistics for Windows (version 20.0, SPSS Inc., Chicago, Ill., USA). The figures were produced using GraphPad Prism (version 6.0.0, GraphPad Software, RRID: SCR_002798).

## Results

### Effect of *C. cicadae* mycelium and *H. erinaceus* Extracts on Neurite Outgrowth in P8 Retinal Explants

**Fig 3:**
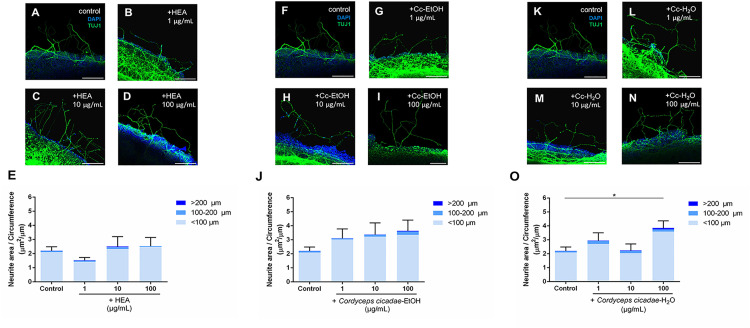
*C. cicadae* mycelium extracts, mainly the aqueous extract, promote neurite outgrowth of retinal explants in P8 mice. (A)-(D) Confocal images of retinal explants from P8 mice cultured without and with N6-(2-Hydroxyethyl) Adenosine (HEA) at concentrations of 1, 10, and 100 μg/mL. TUJ1 (green) shows the neurite outgrowth of retinal explants, and DAPI (blue) is a nuclear marker. (E) HEA did not affect the neurite outgrowth of retinal explants (n = 15 for the control; n = 8 for 1 μg/mL; n = 7 for 10 μg/mL; n = 7 for 100 μg/mL) (F)-(I) Confocal images of retinal explants cultured without and with the ethanol extract of *C. cicadae* (Cc-EtOH) at concentrations of 1, 10, and 100 μg/mL. (J) Cc-EtOH did not affect the neurite outgrowth of retinal explants (n = 15 for the control; n = 6 for 1 μg/mL; n = 6 for 10 μg/mL; n = 6 for 100 μg/mL). (K)-(N) Confocal images of retinal explants cultured without and with the aqueous extract of *C. cicadae* (Cc-H_2_O) at concentrations of 1, 10, and 100 μg/mL. (O) Cc-H_2_O at the concentration of 100 μg/mL enhanced neurite outgrowth of retinal explants (n = 15 for the control; n = 7 for 1 μg/mL; n = 7 for 10 μg/mL; n = 7 for 100 μg/mL). Note: Variability in TUJ1 signal intensity in certain regions likely reflects technical limitations in antibody penetration in areas directly contacting the coverslip, rather than biological variability. Scale bar 100 μm. *p < 0.05; Error bars, mean ± SEM.

**Fig 4:**
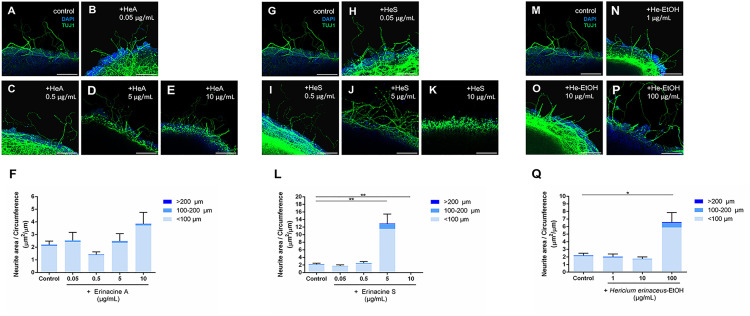
*H. erinaceus* extracts, mainly Erinacine S and the ethanol extract, promote neurite outgrowth of retinal explants in P8 mice. (A)-(E) Confocal images of retinal explants cultured with Erinacine A (HeA) at concentrations of 0.05, 0.5, 5, and 10 μg/mL. TUJ1 (green) shows the neurite outgrowth of retinal explants, and DAPI (blue) is a nuclear marker. (F) Erinacine A did not affect the neurite outgrowth of retinal explants (n = 15 for the control; n = 10 for 0.05 μg/mL; n = 11 for 0.5 μg/mL; n = 8 for 5 μg/mL; n = 6 for 10 μg/mL). (G)-(K) Confocal images of retinal explants cultured with Erinacine S (HeS) at concentrations of 0.05, 0.5, 5, and 10 μg/mL. (L) Erinacine S at the concentration of 5 μg/mL promoted the highest neurite outgrowth in retinal explants, but there was no neurite outgrowth at a concentration of 10 μg/mL. (n = 15 for the control; n = 8 for 0.05 μg/mL; n = 7 for 0.5 μg/mL; n = 7 for 5 μg/mL; n = 7 for 10 μg/mL). (M)-(P) Confocal images of retinal explants cultured without and with the ethanol extract of *H. erinaceus* (He-EtOH) at concentrations of 1, 10, and 100 μg/mL. (Q) He-EtOH at the concentration of 100 μg/mL enhance the neurite outgrowth of retinal explants (n = 15 for the control; n = 10 for 1 μg/mL; n = 8 for 10 μg/mL; n = 8 for 100 μg/mL). Note: Variability in TUJ1 immunostaining intensity in certain areas is attributed to limited antibody penetration at the coverslip interface and does not reflect inconsistency in culture or staining procedures. Scale bar 100 μm. *p < 0.05; **p < 0.01; Error bars, mean ± SEM.

To examine whether the extracts of *C. cicadae* mycelium and *H. erinaceus* are able to promote neurite outgrowth of retinal explants in P8 mice, the various extracts at different concentrations were tested (Fig 3 and Fig 4). Representative images of the wholemount retinal explants are shown in S7 Fig. With respect to the addition of *C. cicadae* mycelium extract, neither HEA nor *C. cicadae*-EtOH showed significant effects in terms of enhancing neurite outgrowth of retinal explants at all concentrations when compared with the control (Fig 3E and 3J). However, *C. cicadae*-H_2_O exhibited a significant enhancement of neurite outgrowth at a concentration of 100 μg/mL when compared with the control, with an average neurite length of 3.85 μm per unit circumference of retinal explant (p = 0.049; Fig 3O). Minor fluctuations were observed at intermediate concentrations (e.g., 10 μg/mL). Only the high concentration of *C. cicadae*-H_2_O significantly promoted neurite outgrowth in retinal explants among the three *C. cicadae* mycelium extracts.

Upon addition of *H. erinaceus* extracts, Erinacine A had no significant effect on promoting neurite outgrowth at all concentrations when compared with the control (Fig 4F). However, both Erinacine S and He-EtOH gave rise to a significant promotion of neurite outgrowth at a concentration of 5 μg/mL, with an average neurite length of 12.94 μm per unit circumference of retinal explant (p = 0.002; Fig 4L) and at a concentration of 100 μg/mL with an average neurite length of 6.60 μm per unit circumference of retinal explant (p = 0.017; Fig 4Q), respectively. Although Erinacine S showed the most prominent effect on neurite outgrowth of retinal explants in this experiment (Fig 4L), there was almost no neurites grown when the concentration of Erinacine S was increased to 10 μg/mL (Fig 4K). Some concentrations showed reduced neurite outgrowth (e.g., 10 μg/mL for Erinacine S), consistent with a biphasic, concentration-sensitive response commonly observed with natural compounds. Other concentrations, such as 0.5 μg/mL for Erinacine A, did not show significant differences compared with controls. This result suggests that a high concentration of Erinacine S might have an opposite effect on the regulation of neurite outgrowth of retinal explants.

To investigate the potential impact of DMSO, which was used as solvent during the preparation of herbal extracts in the present study, the effect of various concentrations of DMSO, ranging from 0.01% to 0.1%, on the neurite outgrowth of retinal explants were examined. The results showed that DMSO at all concentrations did not significantly affect the neurite outgrowth of retinal explants when compared with the control group, which had either no DMSO or alternatively had the DMSO replaced with H_2_O (S3 Fig). These findings suggest that the solvent DMSO does not influence the neurite outgrowth of retinal explants, further confirming that the untreated group is suitable as the negative control.

### Effect of *C. cicadae* and *H. erinaceus* Extracts on Cell Death in Retinal Explants

**Fig 5:**
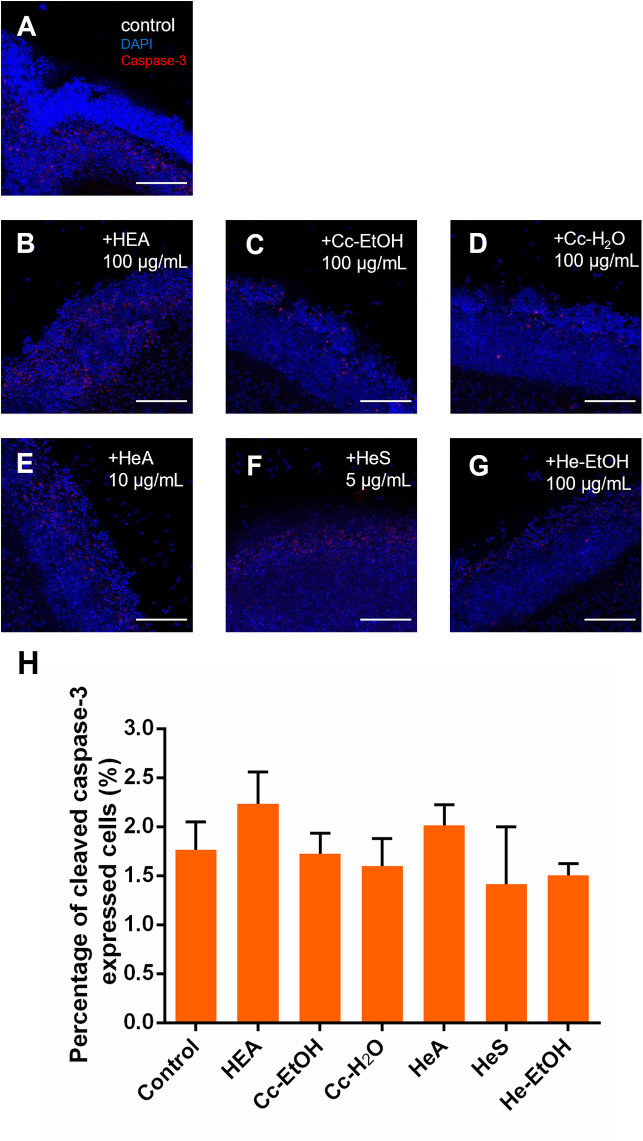
*C. cicadae* and *H.erinaceus* extracts at all concentrations do not affect cell apoptosis of retinal explants. (A)-(G) Confocal images of P8 retinal explants cultured without and with C. cicadae and H. erinaceus extracts, respectively. Retinal explants were immunostained with cleaved caspase-3 (red) and nuclear marker DAPI (blue). (H) N6-(2-Hydroxyethyl) Adenosine (HEA; 100 μg/mL), *C. cicadae*-EtOH (Cc-EtOH; 100 μg/mL), *C. cicadae*-H_2_O (Cc-H_2_O; 100 μg/mL), Erinacine A (HeA; 10 μg/mL), Erinacine S (HeS; 5 μg/mL), and *H. erinaceus*-EtOH (He-EtOH; 100 μg/mL) did not significantly affect the apoptosis of retinal ganglion cells (n = 9 for the control; n = 7 for HEA; n = 8 for CC-EtOH; n = 9 for CC-H_2_O; n = 6 for HeA; n = 7 for HeS; n = 7 for HE-EtOH). Scale bar 100 μm. Error bars, mean ± SEM.

To examine whether the extracts from *C. cicadae* and *H. erinaceus* affect the cell apoptosis of retinal explants, caspase-3, a biomarker associated with apoptosis, was used to assess the number of apoptotic cells via immunostaining. Cells that underwent apoptosis express caspase-3 and show red fluorescence (Fig 5A-5G). The proportion of caspase-3 positive area was used as an index to examine the cell death rate. The concentrations of the extracts were selected based on the optimal concentrations for promoting neurite outgrowth of retinal explants in the present study (HEA 100 μg/mL, Cc-EtOH 100 μg/mL, Cc-H_2_O 100 μg/mL, Erinacine A 10 μg/mL, Erinacine S 5 μg/mL, and He-EtOH 100 μg/mL). The results showed that none of the extracts from *C. cicadae* or *H. erinaceus* had a significant effect on the cell apoptosis of retinal explants (Fig 5H). In addition, DMSO as the solvent also did not influence the cell’s apoptosis of retinal explants (S4 Fig).

### Effect of *C. cicadae* and *H. erinaceus* Extracts on Neurite Outgrowth in P2-4 Retinal Ganglion Cells

**Fig 6:**
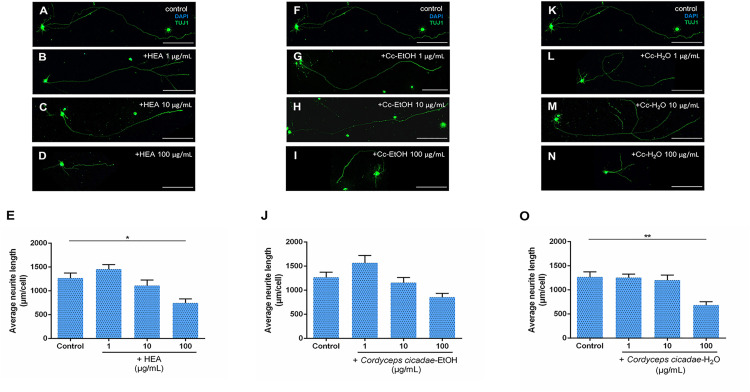
The effects of *C. cicadae* mycelium extracts at different concentrations on neurite outgrowth of retinal ganglion cells in P2-4 mice. **(A)**-**(D)** Confocal images of retinal ganglion cells from P2-4 mice cultured without and with N6-(2-Hydroxyethyl) Adenosine (HEA) at concentrations of 1, 10, and 100 μg/mL. TUJ1 (green) shows the neurite outgrowth of retinal ganglion cells, and DAPI (blue) is a nuclear marker. **(E)** HEA did not significantly promote neurite outgrowth of retinal ganglion cells, rather it inhibited neurite growth at a higher concentration of 100 μg/mL (n = 13 for the control; n = 7 for 1 μg/mL; n = 7 for 10 μg/mL; n = 9 for 100 μg/mL). **(F)**-**(I)** Confocal images of RGCs cultured without and with *C. cicadae*-EtOH (Cc-EtOH) at concentrations of 1, 10 and 100 μg/mL. **(J)** *C. cicadae*-EtOH did not significantly affect neurite outgrowth of RGCs (n = 13 for the control; n = 6 for 1 μg/mL; n = 7 for 10 μg/mL; n = 12 for 100 μg/mL). **(K)**-**(N)** Confocal images of RGCs cultured without and with *C. cicadae*- H_2_O (Cc-H_2_O) at concentrations of 1, 10 and 100 μg/mL. **(O)** *C. cicadae*-H_2_O also had no significant effect on neurite outgrowth of retinal ganglion cells, rather it inhibited neurite growth at a concentration of 100 μg/mL (n = 13 for the control; n = 7 for 1 μg/mL; n = 6 for 10 μg/mL; n = 10 for 100 μg/mL). Scale bar 200 μm. *p < 0.05; **p < 0.01; Error bars, mean ± SEM.

**Fig 7:**
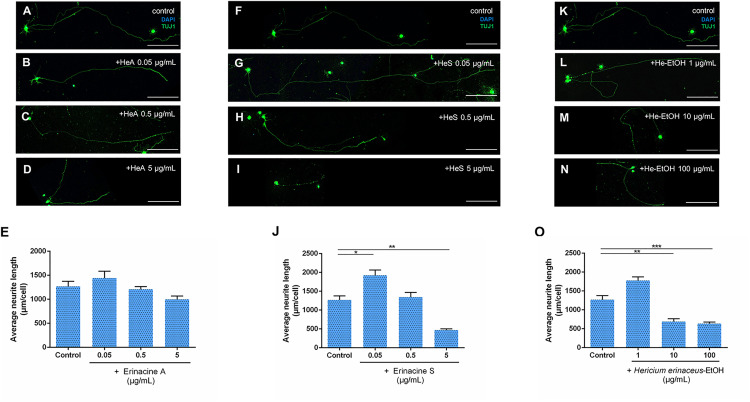
*H. erinaceus* extracts, mainly Erinacine S, promote neurite outgrowth of retinal ganglion cells in P2-4 mice. (A)-(D) Confocal images of retinal explants cultured without and with Erinacine A (HeA) at concentrations of 0.05, 0.5, and 5 μg/mL. TUJ1 (green) shows the neurite outgrowth of retinal ganglion cells, and DAPI (blue) is a nuclear marker. (E) Erinacine A did not significantly affect the neurite outgrowth of retinal ganglion cells (n = 13 for the control; n = 7 for 0.05 μg/mL; n = 8 for 0.5 μg/mL; n = 9 for 5 μg/mL). (F)-(I) Confocal images of retinal ganglion cells cultured without and with Erinacine S (HeS) at concentrations of 0.05, 0.5, and 5 μg/mL. (J) Erinacine S at the concentration of 0.05 μg/mL promoted neurite outgrowth of retinal ganglion cells, but inhibited neurite growth at the concentration of 5 μg/mL. (n = 13 for the control; n = 6 for 0.05 μg/mL; n = 8 for 0.5 μg/mL; n = 11 for 5 μg/mL). (K)-(N) Confocal images of cultured retinal ganglion cells without and with the ethanol extract of *H. erinaceus* (He-EtOH) at concentrations of 1, 10, and 100 μg/mL. (O) *H. erinaceus*-EtOH did not significantly affect the neurite outgrowth of retinal ganglion cells, rather it inhibited neurite growth at the concentrations of 10 μg/mL and 100 μg/mL (n = 13 for the control; n = 6 for 1 μg/mL; n = 7 for 10 μg/mL; n = 11 for 100 μg/mL). Scale bar 200 μm. *p < 0.05; **p < 0.01; ***p < 0.001; Error bars, mean ± SEM.

To examine if the various herbal extracts are able to stimulate neurite outgrowth of RGCs directly, RGCs isolated from the retinas of P2-4 mice were used. In the testing of *C. cicadae* extracts, none of three extracts (HEA, *C. cicadae*-EtOH, and *C. cicadae*-H_2_O) showed a significant effect on the promotion of RGCs neurite outgrowth (Fig 6). Surprisingly, higher concentrations of HEA (100 μg/mL) and *C. cicadae*-H_2_O (100 μg/mL) inhibited RGCs neurite regeneration (Fig 6E and Fig 6O). On testing of *H. erinaceus* extracts, neither Erinacine A nor *H. erinaceus*-EtOH promoted neurite outgrowth of RGCs (Fig 7). Only Erinacine S at a concentration of 0.05 μg/mL showed a significant promotion of RGCs neurite regeneration, with an average neurite length of 1923.11 μm per cell (p = 0.030; Fig 7J). However, both Erinacine S (5 μg/mL) and *H. erinaceus*-EtOH (10 μg/mL and 100 μg/mL) inhibited RGCs neurite outgrowth (Fig 7J and Fig 7O). These results indicate that among the six extracts of *C. cicadae* and *H. erinaceus*, only Erinacine S at the concentration of 0.05 μg/mL has the potential of promoting RGCs neurite outgrowth.

In addition, to investigate the potential impact of DMSO as the solvent during the preparation of herbal extract in the present study, the effect of various concentrations of DMSO ranging from 0.01% to 0.1% on the neurite outgrowth of isolated RGCs were examined. The results showed that DMSO of all concentrations did not significantly affect the neurite outgrowth of RGCs when compared with the control group with either no DMSO or with the DMSO replaced with H_2_O instead (S5 Fig). This finding suggests that the solvent DMSO does not influence the neurite growth of RGCs.

### Effect of *C. cicadae* and *H. erinaceus* Extracts on Neurite Outgrowth in P2-4 Retinal Ganglion Cells

To examine whether the extracts of *C. cicadae* and *H. erinaceus* affect the activity of RGCs neurite outgrowth, the proportion of neurite-bearing cells was used as an indicator of neurite outgrowth activities (S6A Fig). The concentrations of the extracts were selected based on their optimal concentrations for promoting neurite outgrowth of RGCs in the present study (HEA 1 μg/mL, *C. cicadae*-EtOH 1 μg/mL, *C. cicadae*-H_2_O 1 μg/mL, Erinacine A 0.05 μg/mL, Erinacine S 0.05 μg/mL, and *H. erinaceus*-EtOH 1 μg/mL). The results showed that the extracts from *C. cicadae* and *H. erinaceus* did not significantly alter the propotion of neurite-bearing RGCs compared with the control group (S6B Fig). suggesting that these extracts neither promoted nor inhibited the initiation of neurite outgrowth. Notably, although Erinacine S significantly increased the average neurite length (Fig 7J), it did not change the proportion of neurite-bearing cells, indicating that Erinacine S primarily promotes elongation of neurites in already-sprouting RGCs rather than recruiting quiescent cells to initiate neurite outgrowth.

## Discussion

In the present study, the effects of extracts from *C. cicadae* and *H. erinaceus* on retinal explants were examined to determine if these extracts could contribute to the regeneration of RGCs neurites. The results revealed that three extracts, namely *C. cicadae*-H_2_O, Erinacine S, and *H. erinaceus*-EtOH, appeared to facilitate the regeneration of RGC neurites in retinal explants at certain concentrations. However, only Erinacine S was able to significantly promote the neurite outgrowth of isolated RGCs. Therefore, it can be inferred that Erinacine S directly acts on RGCs to stimulate neurite outgrowth, while *C. cicadae*-H_2_O and *H. erinaceus*-EtOH may act indirectly by influencing other retinal cells to facilitate RGCs neurite outgrowth.

### *C. cicadae* and *H. erinaceus* Extracts Are Able to Promote Neurite Outgrowth Rather Than Axon Regeneration in Retinal Explants

In the present experiments, extracts derived from *C. cicadae* and *H. erinaceus* were used. It was observed that *C. cicadae*-H_2_O, Erinacine S, and *H. erinaceus*-EtOH indeed promoted neurite outgrowth of retinal explants (Fig 3 and Fig 4). However, when examining the three regions (“<100 μm”, “100-200 μm”, and “>200 μm”) separately, no significant difference was found between any of the extracts and the control group in terms of neurite length beyond 200 μm. Previous studies have shown that dendrites express both Tau and MAP2 proteins, while axons predominantly express Tau but a lower level of MAP2 protein [34, 35]. In the culture of retinal explants, our previous work has demonstrated that “>200 um” neurites exhibit Tau positivity and MAP2 negativity, suggesting that these long neurites are likely to be axons grown from RGCs [30]. Based on this observation, it can be inferred that these three extracts (*C. cicadae*-H_2_O, Erinacine S, and *H. erinaceus*-EtOH) are able to promote neurite outgrowth of retinal explants but not axon growth.

### The Aqueous Extract of *C. cicadae* promotes neurite outgrowth of retinal explants

It has been reported that extracts from *C. cicadae* can be used to reduce elevated intraocular pressure caused by glaucoma in mice and humans [22, 36]. Additionally, *C. cicadae* extracts have been demonstrated to have antioxidant properties [37]. Based on these findings, it has been hypothesized that *C. cicadae* extracts, with potential to decrease intraocular pressure and exhibit antioxidant effects, may also possess neuroprotection and neuroregeneration properties. In the present study, only the aqueous extract of *C. cicadae* was found to promote neurite outgrowth of retinal explants (Fig 3). It has been known that *C. cicadae*-H_2_O contains a high concentration of adenosine, which may help reduce intracellular oxidative stress and provide neuroprotection [38]. Alternatively, this aqueous extract may contain one or more unknown compounds that acted on other cells in the retina, thereby promoting the neurite outgrowth of retinal explants [36]. In fact, HEA was present in both the aqueous and ethanol extracts. The concentration of HEA in the aqueous extract was 5 mg/g (0.5%), and in the ethanol extract was 3 mg/g (0.3%). Although HEA was present in similar proportions in both extracts, the results showed no significant effect on RGC neurite outgrowth, indicating that HEA may not be related to the neurite outgrowth pathway. Minor reductions in neurite outgrowth were occasionally observed at intermediate concentrations (e.g., 10 μg/mL), but these differences were not statistically significant and likely reflect normal biological variability inherent to explant cultures rather than true inhibitory effects.

### Both Erinacine S and Ethanol Extract of *H. erinaceus* Promote Neurite Outgrowth of Retinal Explants, While Only Erinacine S Facilitates Axon Growth of RGCs Directly

It has been demonstrated that Tau and MAP2 are reliable markers for confirming that the growing neurites of isolated RGCs are axons or dendrites [39]. Specifically, axons are identified as Tau-positive with a thinner and elongated morphology, while dendrites are MAP2-positive with a thicker and shorter morphology. Therefore, the increased average neurite length observed in isolated RGCs suggests enhanced axon elongation. This interpretation is supported by previous morphological studies showing that longer RGC neurites correlate with Tau-positive axons, although direct axonal marker staining was not performed in the present study.

Recent studies have also shown that *H. erinaceus* is able to contribute to myelin generation and maturation of oligodendrocytes [28]. Additionally, it has been found that *H. erinaceus* is able to reduce oxidative stress and provide neuroprotection in the mouse model of Alzheimer’s disease [40]. In the present study, both Erinacine S and an ethanol extract of *H. erinaceus* were found to promote neurite outgrowth of retinal explants (Fig 4). However, only Erinacine S directly acted on RGCs to stimulate neurites regeneration (Fig 7). Previous research has indicated that the ethanol extract of *H. erinaceus* and erinacine contributed to an increase in the synthesis of nerve growth factor (NGF) in cells [27, 41]. It is possible that *H. erinaceus*-EtOH is able to act on other types of cells in the retina, which results in the release of NGF, which promotes the neurite outgrowth of retinal explants. Furthermore, previous studies have shown that Erinacines A to I are able to enhance NGF synthesis, thereby promoting neurite regeneration. Thus, distinct erinacines might have a range of effects on different cell types [42]. Additionally, the efficacy of Erinacines might not be related to their proportions in the extract. The concentration of Erinacine A in the *H. erinaceus* extract was 28.3 mg/g (2.83%), and the concentration of Erinacine S was 5.8 mg/g (0.58%). Despite Erinacine A being more abundant in the extract compared to Erinacine S, the results showed that Erinacine S was more effective in promoting RGC neurite regeneration. Although the function of Erinacine S has not been well studied, considering the structural similarity among all the Erinacines A to S compounds, it may be inferred that Erinacine S also possesses the capacity to enhance NGF synthesis, or bind to specific receptors in the cells, triggering downstream reactions that promote neurite outgrowth and thereby promoting RGCs neurite outgrowth.

Interestingly, both Erinacine S and HEA made up less than 1% of their respective extracts. However, only Erinacine S was effective in RGC neurite regeneration, suggesting that the proportion of a compound in an extract does not directly influence its cellular effects. Occasional non-linear responses were observed at intermediate or high concentrations (e.g., 0.5 μg/mL or 10 μg/mL Erinacine S), where neurite outgrowth was reduced compared with lower concentrations. In particular, the reduction at 10 μg/mL was statistically significant, suggesting a narrow therapeutic window and potential dose-dependent inhibitory effects at higher concentrations. Such biphasic responses are consistent with previous reports of concentration-sensitive activities of natural compounds.

### *C. cicadae* and *H. erinaceus* Do Not Affect Cell Apoptosis and Neurite Outgrowth Activity in Retinal Explants

In the present study, it was found that none of the extracts from *C. cicadae* or *H. erinaceus* affect the apoptosis of RGCs in retinal explants (Fig 5). Other studies on *C. cicadae* have shown that its extracts are able to reduce intracellular oxidative stress, decrease cell apoptosis, and provide neuroprotection [43]. When treating cancers, clinical drugs tend to induce reactive oxygen species (ROS) in cells and increase cell apoptosis and in this context administering *C. cicadae* extract has been shown to reduce ROS and apoptosis in cells [44]. Thus *C. cicadae* extracts have a potential of reducing oxidative stress without affecting cell apoptosis at appropriate concentrations.

Regarding *H. erinaceus*, previous studies have shown that its extracts not only promote NGF synthesis for neurite regeneration, but also reduce oxidative stress [45, 46]. In a separate experiment, it has been found that *H. erinaceus* extract is able to effectively mitigate H_2_O_2_-induced oxidative damage [47]. These studies demonstrate that *H. erinaceus* extracts also have the potential to reduce oxidative stress without affecting cell apoptosis at appropriate concentrations. Furthermore, it has been reported that *H. erinaceus* is able to promote neurite outgrowth activity and increase the number of neurite-bearing cells [48]. Interestingly, in our experiments, the extracts from *C. cicadae* and *H. erinaceus* had no effect on the number of neurite-bearing RGCs (S6 Fig). This suggests that *H. erinaceus* may have a cell type specific effect.

### Potential of *C. cicadae* in Terms of Neuroregeneration and Neuroprotection

Most of the current research on *C. cicadae* has primarily focused on reducing cellular oxidative stress and preventing apoptosis, and only a few studies have focused on neuroregeneration. Studies on *Cordyceps* spp. in traditional Chinese medicine mainly have emphasized the effectiveness of Chinese caterpillar fungus or “Dong Chong Xia Cao” [49]. One report has indicated that *C. cicadae* has similar biochemical characteristics to *Cordyceps sinensis* and *Cordyceps militaris* [50]. Extracts from *C. sinensis*, *C. militaris*, and other *Cordyceps* spp. contain cordycepin, which is a derivative of adenosine [51, 52]. *Cordyceps* spp. produce cordycepin when infecting larvae or pupae, and it has been observed that cordycepin promotes the growth of *C. militaris* in larvae [53]. Cordycepin acts on the adenosine 1 receptor (A1R) by reducing cyclic adenosine monophosphate (cAMP) and protein kinase A (PKA) levels. When PKA expression decreases, this inhibits the expression of downstream inflammatory factors. As a result, cordycepin modulates synaptic plasticity and prevents the formation of excessive ROS in neuronal cells. These properties make cordycepin neuroprotective [54]. If *C. cicadae* also contains cordycepin or a similar compound, or other derivatives of adenosine, its molecular mechanism may also involve action on A1R in order to achieve neuroprotection.

In the present study, the aqueous extract of *C. cicadae* may act on Müller glia cells in the retina, which will induce the release of superoxide dismutase 2 (SOD2), catalase (CAT), or NGF [55], thereby stimulating RGCs and achieving neuroprotection and neuroregeneration. Future studies should investigate the various components present in *C. cicadae*-H_2_O and examine whether these components affect retinal Müller glia cells, leading to the release of antioxidant enzymes or NGF, and the promotion of RGC neurite outgrowth. It is also important to study how these components interact with receptors on retinal cells and activate specific pathways to facilitate neuroprotection and RGC neurite outgrowth.

### Potential of *H. erinaceus* For Neuroregeneration

Many studies have used extracts of *H. erinaceus* to tackle neurological diseases such as Alzheimer’s disease, Parkinson’s disease, and spinal cord injury. For example, an extract from *H. erinaceus* has been shown to increase the production of neurotrophic factors in cells [56, 57]. In the present study, the ethanol extract of *H. erinaceus* did not have a direct effect on RGC neurite outgrowth but rather showed a promotion of neurite regeneration of RGCs in retinal explants (Fig 4Q and Fig 7O). This suggests that the components of *H. erinaceus*-EtOH may stimulate retinal Müller glia cells to release NGF or alternatively activate other retinal cells to release neurotrophic factors, thereby promoting RGC neurite outgrowth.

Erinacines, the compounds from the ethanol extract of *H. erinaceus* is much more important than ergothioneine, the compound from the water extract of *H. erinaceus*. This is the main reason that the present study focused on erinacines. Erinacine A has been found to promote NGF production, and NGF is known to activate tyrosine kinase A (TrkA) and extracellular signal-regulated protein kinase 1/2 (Erk1/2) to regulate neurite outgrowth in neuronal cells [58]. However, different erinacines have distinct effects in different cells. In our experiment, Erinacine A did not promote neural regeneration (Fig 4F and Fig 7E), but it is assumed that Erinacine S may promote nerve regeneration through the TrkA/Erk1/2 pathway in the present study. It is well known that the main factors that regulate axon regeneration of RGCs include Elk-1, PTEN, and REST [59]. Specifically, Elk-1, a transcriptional activator, is able to regulate RGC neurite outgrowth and has neuroprotective functions [60]. PTEN, a negative regulator of the PI3 kinase-Akt signaling pathway, is known to inhibit Elk-1 expression [61]. REST, a repressor element 1-silencing transcription factor, has also been reported to inhibit downstream functions of Elk-1 [62]. Based on these previous studies, it is hypothesized that Erinacine S could inhibit the expression of PTEN or REST, which may then lead to Elk-1 expression and activation of the downstream genes related to RGC neurite outgrowth. In addition, a recent study has shown that Erinacine S is able to promote neurite regeneration of dorsal root ganglion neurons and cortical neurons in rats, and this was through stimulating the production of neurosteroids, which are able to facilitate neurite outgrowth and protect neurons against apoptosis [32]. Moreover, another study has found that retinal amacrine cells and retinal ganglion cells possess the capacity to synthesize neurosteroids [63]. Certain types of neurosteroids are able to interact with nuclear receptors, modulate gene transcription, exert positive effects on NMDA receptors and inhibit GABA_A_ receptors, leading to the promotion of neuroplasticity and synaptogenesis in neuronal cells [64]. Therefore, it is postulated that Erinacine S may increase the production of neurosteroids in RGCs, leading to RGC neurite outgrowth. Since the ability of Erinacine S to promote neuroregeneration has been demonstrated and it has been shown to cross the blood-brain barrier [29], further research should be conducted to determine how effective it is at crossing the blood-retinal barrier. If confirmed, this would be extremely beneficial for the development of oral medications or health supplements in the future.

### Limitations and future directions

One limitation of this study is the use of mice at different postnatal ages in the two experimental setups: postnatal day 8 (P8) mice for the retinal explant experiments and neonatal mice (P2-4) for the isolated RGCs cultures. P8 retinal explants were selected to better reflect the structural and cellular complexity of the in vivo retinal environment, where multiple retinal cell types may interact and influence neurite outgrowth. Conversely, isolated RGC cultures from P2-4 mice were used to assess the direct effects of the extracts on RGCs in a simplified system, minimizing confounding influenced from other retinal cell types. Nevertheless, we acknowledge that differences in developmental stage limit direct comparability between these two models and may reduce the strength of conclusions regarding a unified neuroregenerative mechanism. Accordingly, the results should be interpreted as complementary observations obtained from distinct experimental contexts rather than definitive evidence supporting a single hypothesis.

Additionally, the scarcity of research on the specific extracts we used, particularly concerning their potential to promote retinal neuron regeneration, limits our understanding of the underlying mechanisms. As a result, the precise mechanisms through which Erinacine S influences neurite outgrowth, and its detailed intracellular pathways, remain unclear. The complex composition of the *C. cicadae*-H_2_O and the *H. erinaceus*-EtOH makes it challenging to identify the specific components responsible for promoting retinal explant neurite regeneration. Furthermore, unknown cellular targets of Erinacine A, Erinacine S, and HEA, as well as unverified signaling pathways of activation or inhibition of these compounds in the targeted cells, also limit the understanding of their specific effects. Nevertheless, these extracts may first affect other retinal cells, then subsequently influence RGCs neurite growth.

Our findings demonstrated that *C. cicadae*-H_2_O, Erinacine S, and *H. erinaceus*-EtOH promoted general neurite outgrowth in retinal explants, while Erinacine S promoted neurite growth in isolated RGCs. However, it is important to note that since we did not perform Tau and MAP2 staining in the present study, it is not known if these grown neurites are dendrites or axons. This also highlights a limitation of this study. Finally, as with all in vitro studies, the results must be interpreted cautiously. The demonstration of a narrow therapeutic window for potent agents like Erinacine S, where high concentrations become inhibitory, mandates careful dose-ranging and safety assessment during future in vivo translational studies.

Another limitation is that the experiments were conducted in vitro. Although certain extracts at specific concentrations demonstrated the ability to promote retinal explant and RGCs neurite growth, higher concentrations resulted in neurite growth inhibition, suggesting potential toxicity. This indicates that careful dosing and concentration control will be essential for animal studies or therapeutic applications in the future. Positive controls, such as PTEN knockdown or BDNF could be done in the future study for the better demonstration of the experimental models in this study.

Differences in physiological mechanisms potentially induced by aqueous versus ethanol extracts are worth studied in the future. The polarity of the extraction solvent may influence the spectrum of bioactive compounds obtained, and different solvents may affect drug transportation in tissue and cells, further affects their effects on RGCs neurite outgrowth. We could know the effects of extraction to RGCs from this study, but the physiological and biological mechanisms remain unknown. The molecular mechanistic experiments could be done in the future. For instance, western blotting for NGF, TrkA/Erk1/2, or PTEN/REST pathways. These experiments could decipher potential pathways about Erinacine S’s affection on RGCs. Especially identification of Erinacine S is the highlight of this study, so the future study should focus on the mechanism and in vivo efficacy of Erinacine S. Furthermore, future studies should consider conducting in vivo experiments to better understand the therapeutic potential and safety of these extracts in a more complex biological environment. Also, future studies should include direct RGCs survival analysis because it is important to show cytotoxicity of extractions for the future applications. Testing extractions effect to RGCs on glaucoma pathologic model is another cutting point in the future. Additionally, exploring the use of human iPSCs to generate retinal neurons could provide insights more relevant to human medicine and health care applications. Investigating the potential synergistic effects of combining these extracts with other known neuroprotective agents might also offer a more comprehensive approach to promoting retinal neuron regeneration.

## Supporting information

S1 Fig HPLC analysis comparing *C. cicadae* ethanol extract and aqueous extract with the HEA standard. (TIF)

S2 Fig HPLC analysis comparing *H. erinaceus* ethanol extract with HeA and HeS standards. (TIF)

S3 Fig The solvent DMSO does not affect neurite outgrowth of retinal explants in P8 mice. (TIF)

S4 Fig The solvent DMSO does not affect cell survival of retinal explants. (TIF)

S5 Fig The solvent DMSO does not affect neurite outgrowth of retinal ganglion cells in P2-4 mice. (TIF)

S6 Fig *C. cicadae* and *H. erinaceus* extracts do not affect neurite outgrowth activities of retinal ganglion cells in P2-4 mice. (TIF)

S7 Fig Neurite outgrowth in wholemount retinal explants treated with different agents. (TIF)
