# Supplementary figures and images for: Extracts from Cordyceps cicadae and Hericium erinaceus promote the neurite outgrowth of retinal ganglion cells
Source: PLoS One. 2026 Feb 6;21(2):e0342244. doi: 10.1371/journal.pone.0342244 (PMC12880703; doi:10.1371/journal.pone.0342244)

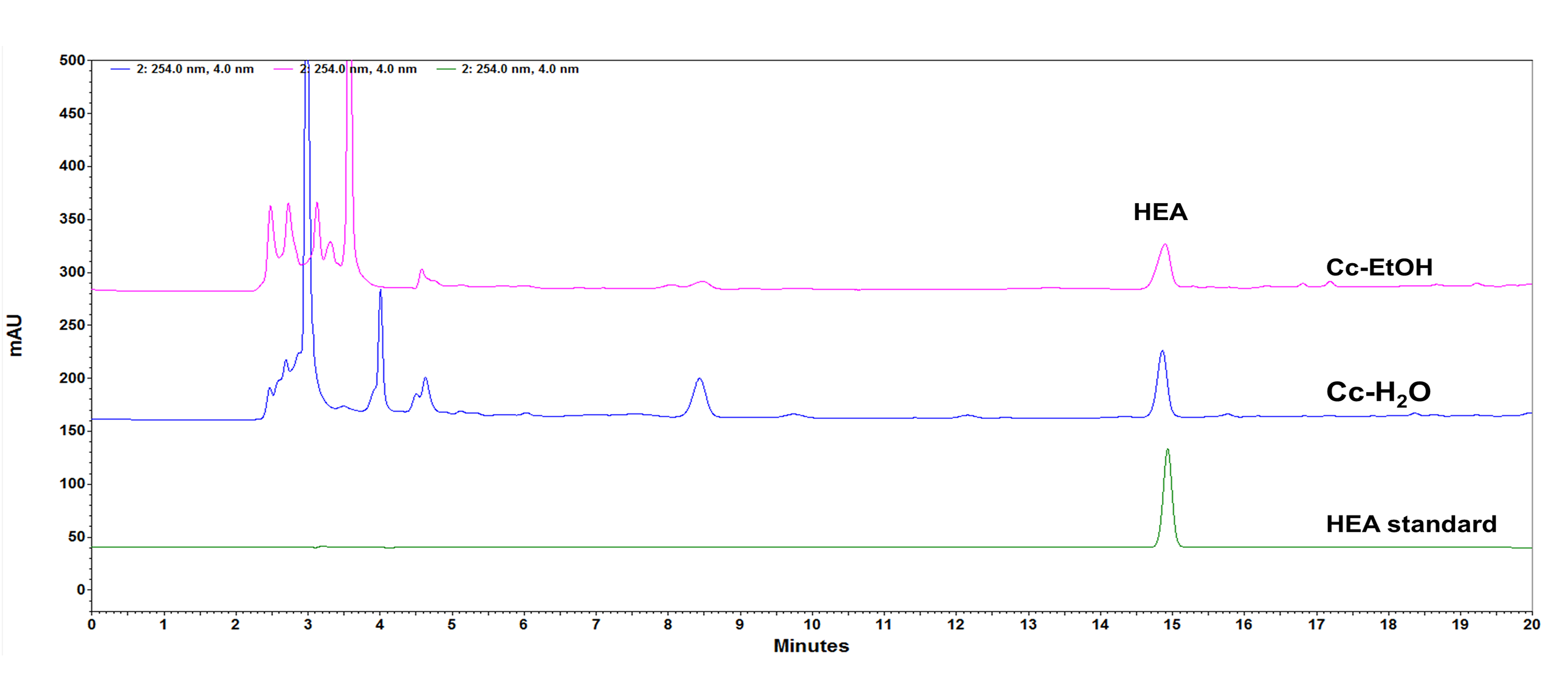

Supplement: S1 Fig — (TIF) [file pone.0342244.s001.tif]

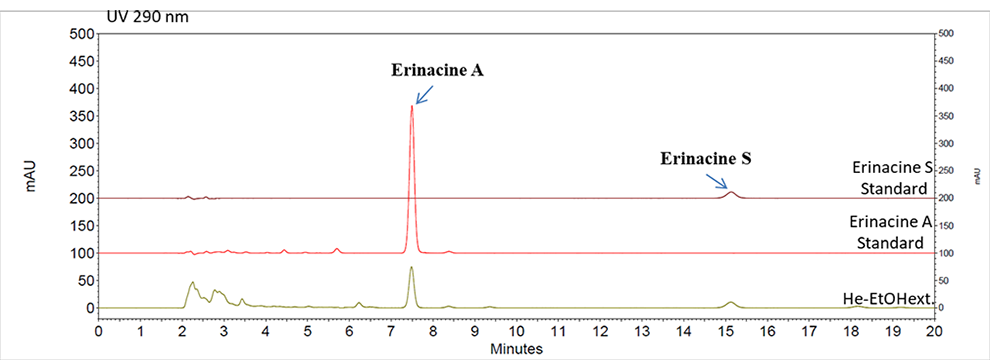

Supplement: S2 Fig — (TIF) [file pone.0342244.s002.tif]

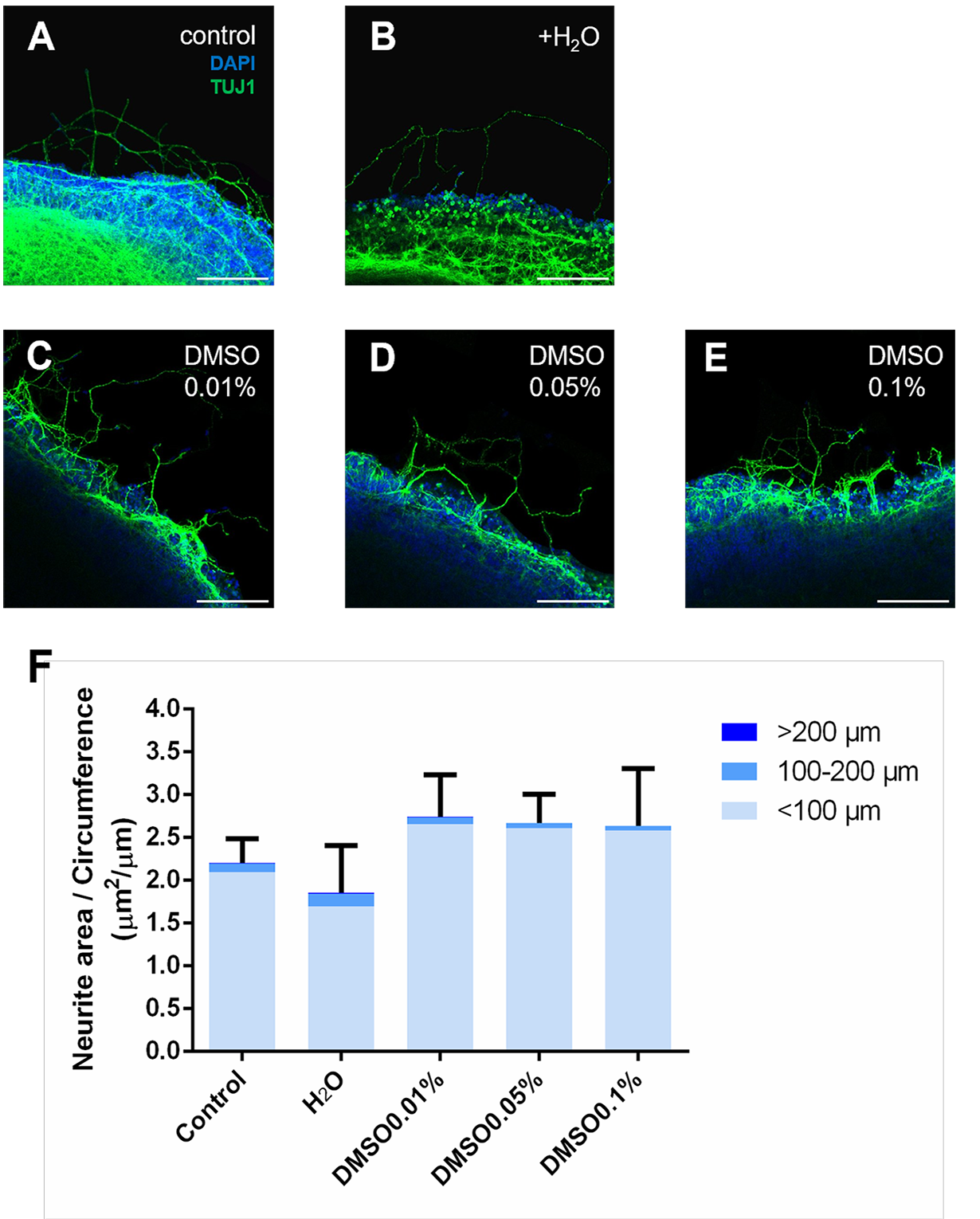

Supplement: S3 Fig — (TIF) [file pone.0342244.s003.tif]

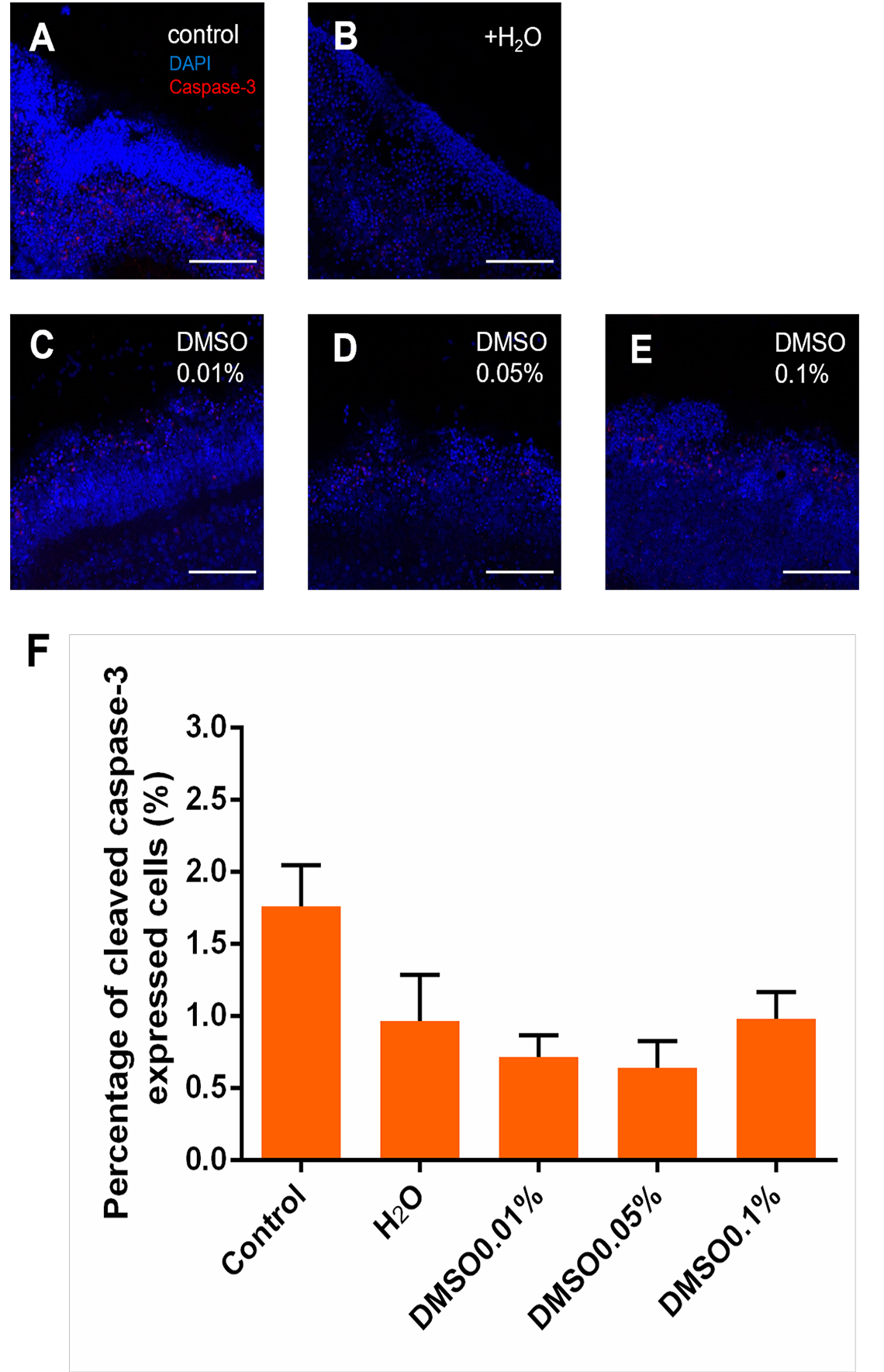

Supplement: S4 Fig — (TIF) [file pone.0342244.s004.tif]

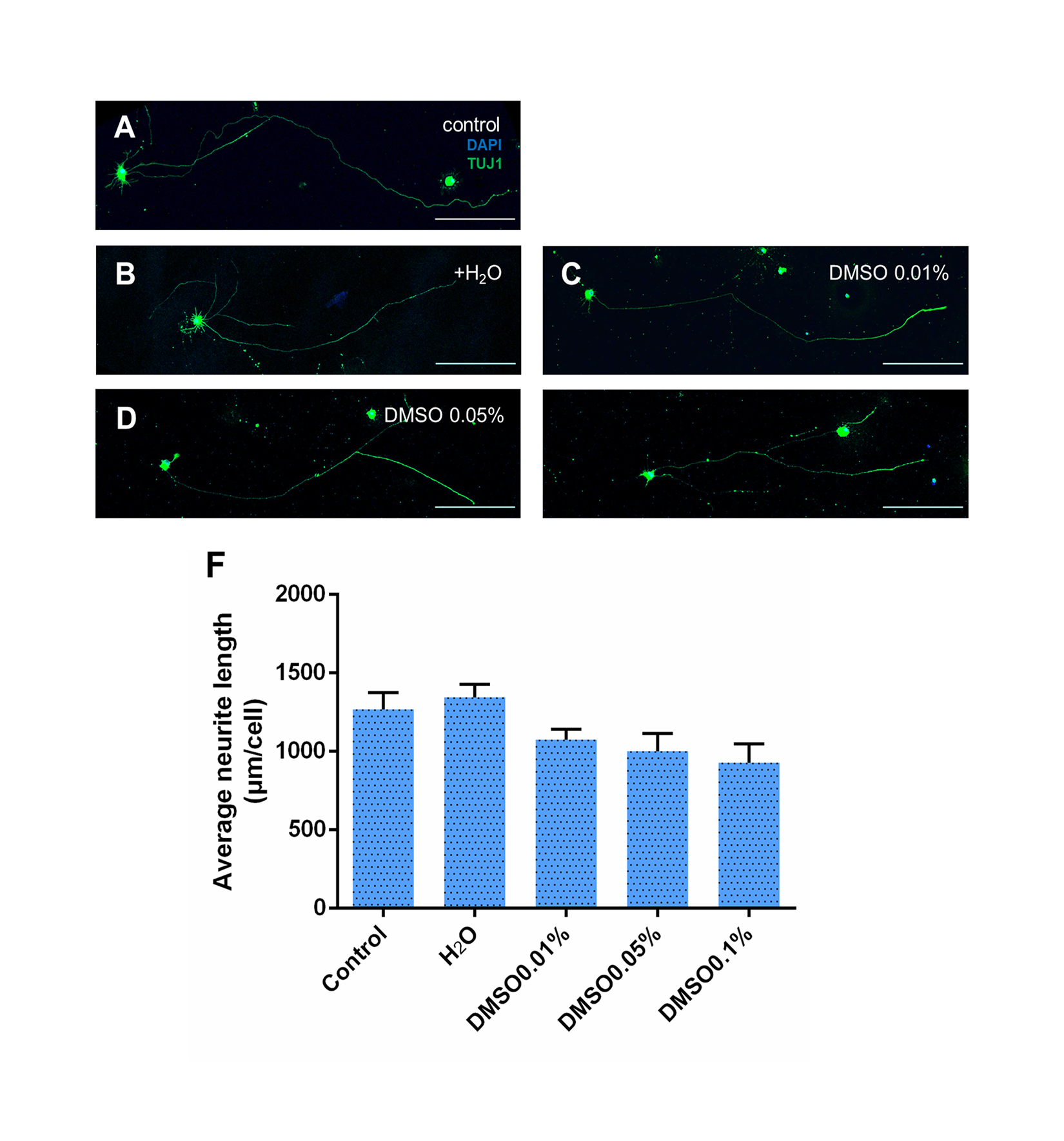

Supplement: S5 Fig — (TIF) [file pone.0342244.s005.tif]

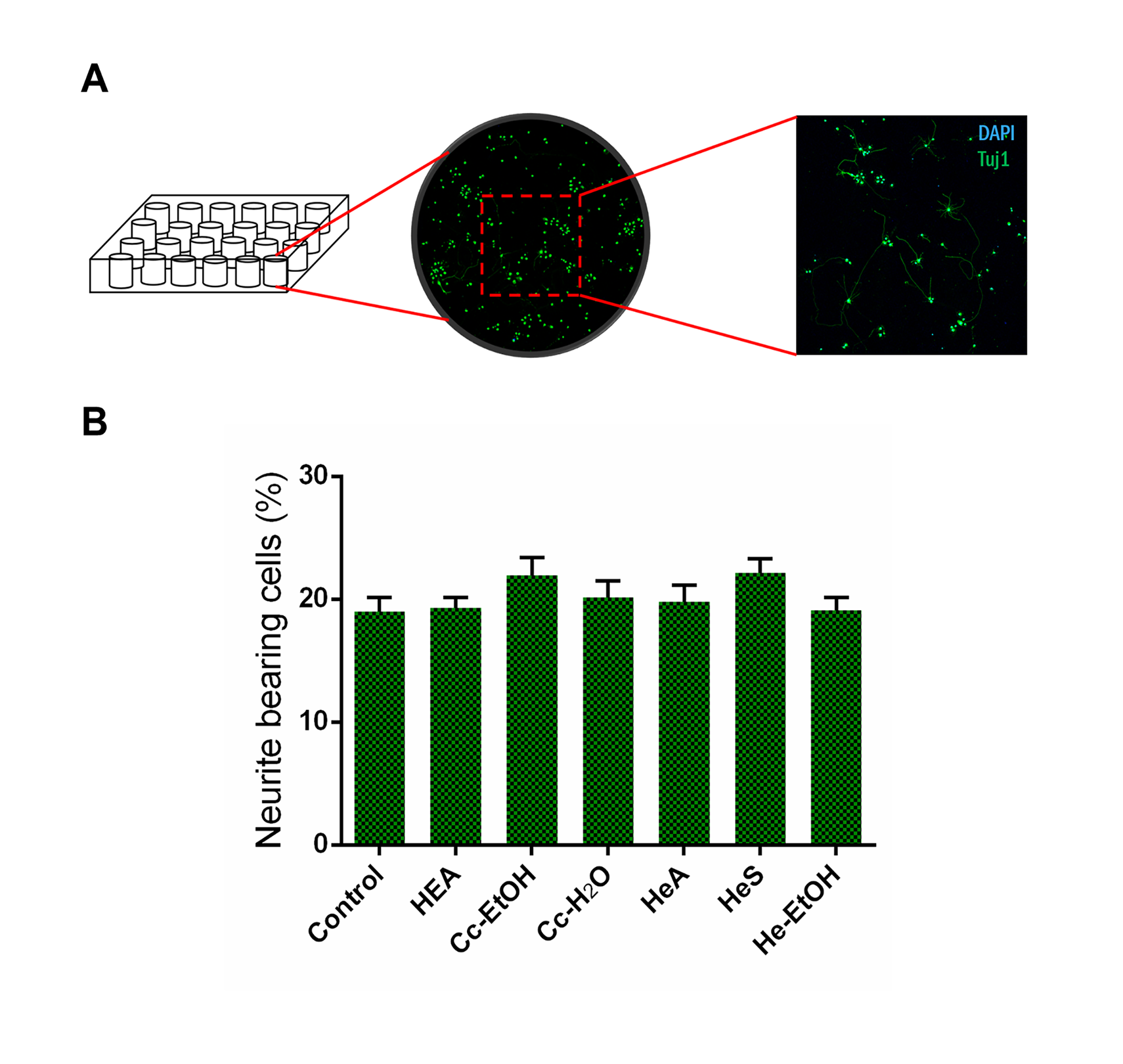

Supplement: S6 Fig — (TIF) [file pone.0342244.s006.tif]

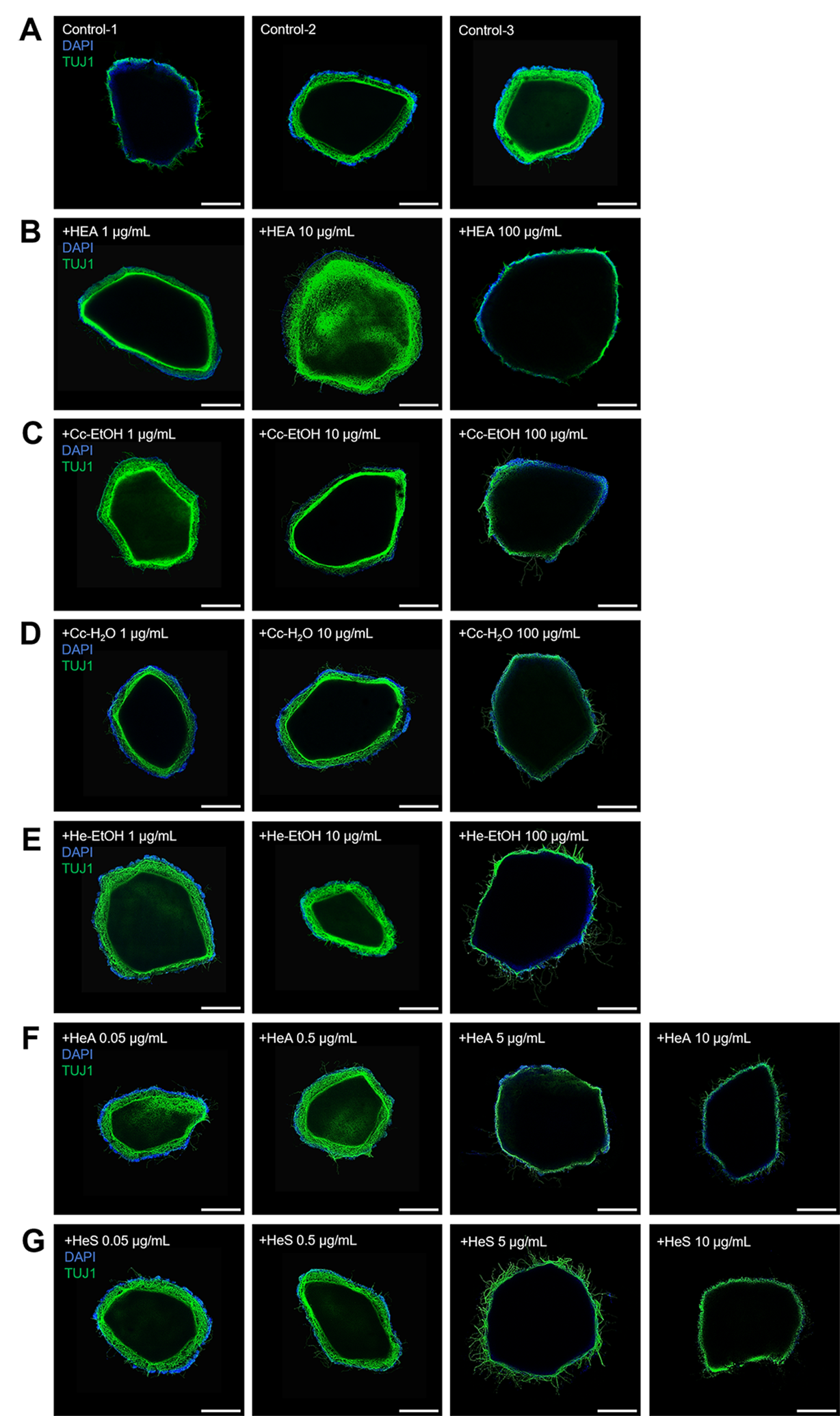

Supplement: S7 Fig — (TIF) [file pone.0342244.s007.tif]
